# A novel mutation in *COL3A1* associates to vascular Ehlers–Danlos syndrome with predominant musculoskeletal involvement

**DOI:** 10.1002/mgg3.1753

**Published:** 2021-07-28

**Authors:** Federica Ruscitti, Lucia Trevisan, Giulia Rosti, Fabio Gotta, Annalia Cianflone, Alessandro Geroldi, Paola Origone, Anna Pichiecchio, Simona Viglio, Maria Iascone, Paola Mandich

**Affiliations:** ^1^ DINOGMI Department University of Genoa Genoa Italy; ^2^ Medical Genetics Unit IRCCS Ospedale Policlinico San Martino Genoa Italy; ^3^ Neuroradiology Department IRCCS Mondino Foundation Pavia Italy; ^4^ Department of Brain and Behavioral Sciences University of Pavia Pavia Italy; ^5^ Biochemistry Unit Department of Molecular Medicine University of Pavia Pavia Italy; ^6^ Medical Genetics Laboratory Hospital Papa Giovanni XXIII Bergamo Italy

**Keywords:** *COL3A1*, musculoskeletal involvement, NGS, vEDS

## Abstract

**Background:**

Vascular Ehlers–Danlos syndrome (vEDS) is a heritable connective tissue disorder caused by defects in the type III collagen protein. It is generally considered the most severe form of Ehlers–Danlos syndrome (EDS) due to an increased risk of spontaneous artery or organ rupture. vEDS has an extremely heterogeneous presentation and muscle rupture is considered a minor diagnostic criterium.

**Methods:**

A patient with a long history of inconclusive examinations and investigations was referred to our unit. The clinical picture was mainly characterized by muscle ruptures, whereas the cardiovascular involvement was limited to mitral regurgitation. We performed a panel analysis of genes associated with inheritable heart diseases using the TruSight Cardio kit (Illumina). A skin biopsy was then performed for functional studies to analyze the different forms of collagen molecules produced in vitro by cutaneous fibroblasts.

**Results:**

The patient presented the novel variant c.3478A>G (p.Ile1160Val) in *COL3A1* (NM_000090.3), whose pathogenicity was supported by biochemical analysis of type III collagen.

**Conclusion:**

In this report, we describe a case of vEDS with predominant and severe musculoskeletal involvement. Our findings provide insight into genetic variants and clinical expression of vEDS, broadening the clinical scenario of the syndrome.

## Graphical abstract

In this report, we describe a case of vEDS with predominant and severe musculoskeletal involvement. Our findings provide insight into genetic variants and clinical expression of vEDS, enlarging the clinical scenario of the syndrome.

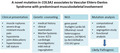

## 1 INTRODUCTION

*COL3A1* (OMIM *120180) encodes for collagen pro‐α1(III) chain, also known as pro‐α1 chain of type III collagen. Pro‐α1 chains are hydroxylated, glycosylated and incorporated into trimers to form the procollagen. This molecule undergoes secretion and removal of his amino‐terminal and carboxy‐terminal ends to form tropocollagen fibers, whose association leads to the production of collagen fibers. Three α1 chains are included in a type III collagen molecule, which has a long triple‐helical domain (Kuivaniemi & Tromp, 2019). Type III collagen constitutes about 5–20% of the entire collagen content in the human body (Miller, 1988). It plays an essential role in the structural integrity of arteries, uterus, and bowel (Byers, 1999 [updated 2019]; Byers et al., 2017; Malfait et al., 2017; Malfait, 2018) and it has been also detected in skin, tendons, ligaments, and bones (Keene et al., 1991).

Heterozygous mutations in *COL3A1* are associated with the vascular type of Ehlers–Danlos syndrome (vEDS). Ehlers–Danlos syndrome (EDS) is a heterogeneous group of heritable connective tissue disorders. The phenotype is variable and mainly comprises joint hypermobility, skin hyperextensibility, and tissue fragility. EDS is classified in 13 different subtypes. Among these, the vascular type is the most severe and life‐threatening due to vascular dissection, gastrointestinal perforation, or organ rupture. vEDS can also be characterized by spontaneous uterine rupture, carotid‐cavernous sinus fistula in the absence of trauma, bruising disproportionate to trauma, thin and translucent skin, spontaneous pneumothorax, acrogeria, clubbed foot, congenital hip dislocation, hypermobility of small joints, tendon and muscle rupture, keratoconus and gingival recession, and fragility (Malfait et al., 2017).

Due to the heterogeneous clinical presentation, the diagnosis of vEDS may be challenging. Therefore, it can be established through either the identification of a heterozygous causative variant in *COL3A1* or the detection of abnormal synthesis and mobility of type III collagen chains on biochemical analysis of type III collagen from cultured fibroblasts (Byers, 1999 [updated 2019]).

We hereby report a novel *COL3A1* variant causing vEDS with predominant and severe musculoskeletal involvement. Our findings provide insight into genetic variants and clinical expression of vEDS, broadening the clinical vEDS scenario.

## 2 CASE REPORT

The proband is a 35‐year‐old male with a 10‐year history of sprains and muscle ruptures. He reported six left knee sprains since the age of 17 and two ruptures of the right rectus femoris muscle at the age of 29 and 30, respectively. The patient did not mention any strenuous activity that could explain the ruptures. Given the presence of weakness and discomfort in the shoulder girdle, an ultrasonography of the left superior arm was performed, showing calcifications of the tendon of the supraspinatus muscle.

At the age of 31, the patient reported a rupture of the right biceps femoris muscle that occurred in the attempt to dismount his motorcycle. These episodes were followed by a series of spontaneous muscle ruptures of the right biceps femoris and other muscles not related to any exertion.

At the age of 30, the patient started complaining about generalized muscle pain and progressive hyposthenia in his proximal lower limbs. The x‐ray of the hip bones was normal. The neurological examination and the electrophysiological studies of the limbs were negative. A spinal MRI revealed several disc herniations for which the patient had undergone surgery, without pain relief. Since the muscular pain was invalidating, an opioid therapy was started leading to partial remission.

Due to the lack of an underlying diagnosis, even after a series of clinical evaluations, the patient came to our attention.

On physical examination, the proband was slender and considerably taller than his parents (185 cm vs. 160 cm of the mother and 161 cm of the father). He reported a growing spurt at the age of 16, with a gain of 22 cm in 1 year (from 160 to 182 cm of height). Other findings were pectus excavatum, arm span of 187 cm (arm span/height ratio: 1.01), flat feet, and cutaneous striae on the hips and the thighs.

On the clinical suspicion of Marfan syndrome, the Marfan Systemic Score (Dietz, 2001 [updated 2017]) was assessed and a score of 4/23 was obtained, making this diagnosis unlikely. Moreover, the ocular examination was normal and the ultrasound of the abdomen did not reveal any vascular abnormality. Serum IGF1 levels, assayed to rule out a pituitary adenoma as the cause of the patient's growing spurt, was normal.

Further examinations were performed to investigate the cardiovascular system. An ECG revealed right branch block and left branch hemiblock with a right axis deviation (−120°). An echocardiography showed mild mitral regurgitation and delayed diastolic relaxation. These findings were confirmed during the following 3 years of cardiologic follow‐up, without significant variations.

Vascular echo‐Doppler showed normal structure and diameters of the aorta and the supra‐aortic trunks, as well as of the upper and lower limbs’ arteries.

After coming to our attention, the patient started presenting paroxysmal hypertensive episodes. Further workup, aimed at ruling out pheochromocytoma, revealed normal plasma and urinary metanephrines and no pathologic accumulation of fluorodeoxyglucose (FDG) at the PET scan examination. Repeated Holter monitoring blood pressure showed that daytime values of heart rate and blood pressure (bp) were set at the upper normal levels and there was a significant variability in daytime blood pressure rates with a physiological reduction at night (this pattern is defined as “dipper status”). One year later the variation between daytime and nighttime bp levels further increased (i.e., “extreme dipper”). A therapy with lisinopril failed to guarantee a complete pressure control, so the patient was referred to cardiological follow‐up. A switch to carvedilol caused the onset of faintings (min bp: 40 mmHg), so lisinopril was reintroduced. The opioid therapy for chronic pain is still ongoing.

Although the family history was totally negative for collagen‐related diseases, the recurrence of sprains and the evidence of cutaneous striae on clinical examination were suggestive of a collagenopathy with cardiac involvement.

Therefore, we considered the possibility of a cardiac‐valvular form of EDS, so we performed a panel analysis of genes associated with inheritable heart diseases. Genomic DNA was extracted from peripheral blood samples using standard procedure. Briefly, the exonic and flanking splice junction regions of genes of interest were captured using the TruSight Cardio kit (Illumina) and sequenced on a MiSeq Illumina system with 150bp paired‐end reads. Reads were aligned to human genome build GRCh37/UCSC hg19 and analyzed for sequence variants using a custom‐developed analysis tool (Sana et al., 2016), selecting collagenopathy genes to create a virtual panel. Mean coverage was 285x and >30x for each target region.

On note, *FBN1* gene was included in the panel and was normal, furtherly disproving the initial suspect of Marfan syndrome. The analysis showed the presence of the heterozygous variant c.3478A>G (p.Ile1160Val) in *COL3A1* (NM_000090.3).

This variant has never been reported neither in literature nor in the major genetic databases (gnomAD, ExAC). Following ACMG2015 criteria, the mutation was classified as a variant of uncertain significance. It is predicted as neutral by some bioinformatics tools (Provean, Polyphen 2, Mutation Assessor and Mutpred) and damaging by others (SIFT, Mutation Taster and CADD).

**FIGURE 1:**
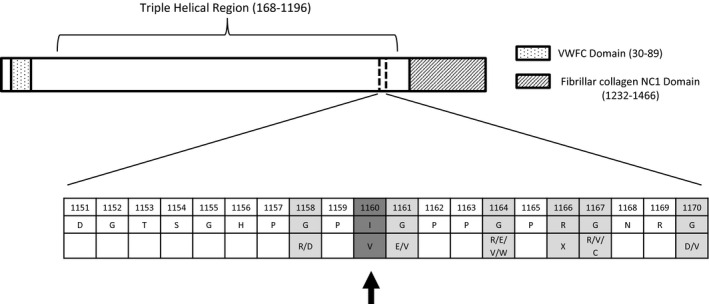
Schematic representation of collagen α1(III) chain: a 10‐amino acid regions upstream and downstream p.Ile1160Val is focused in detail to show all the nearby mutations

**FIGURE 2:**
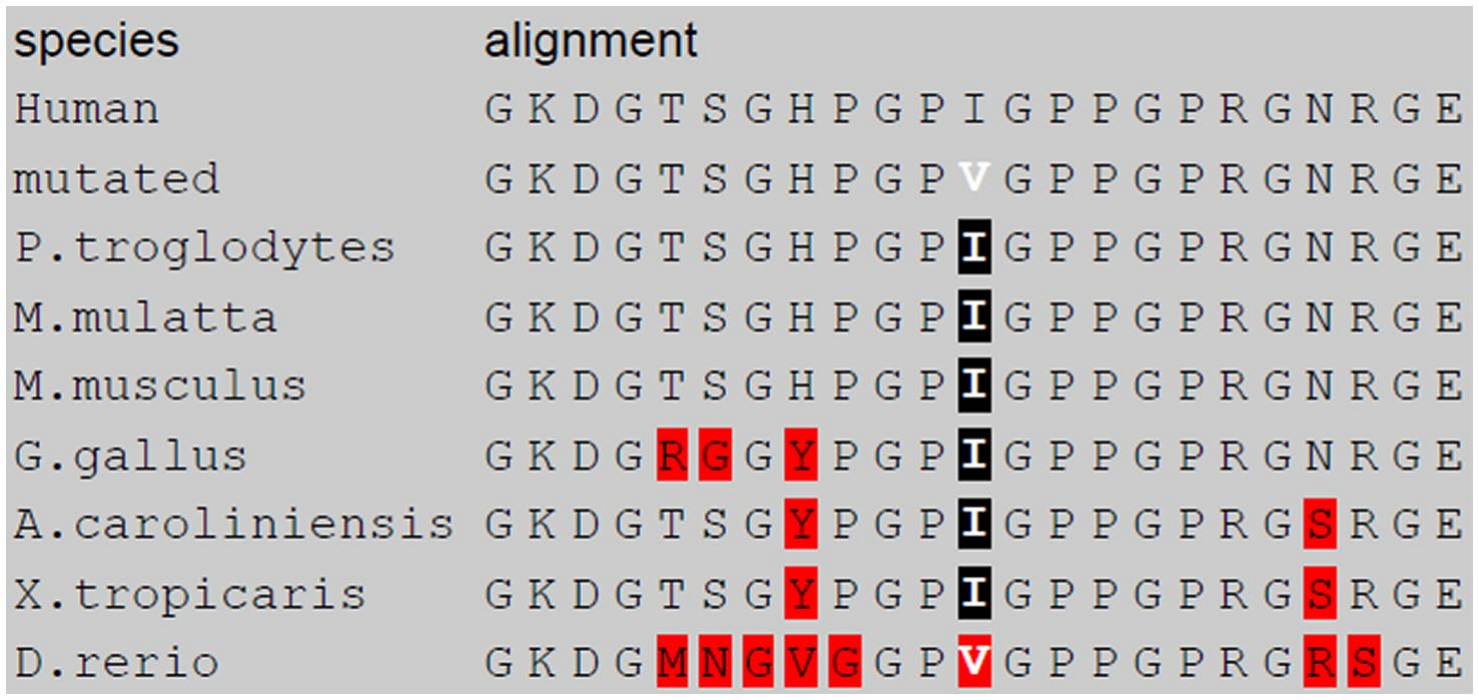
Isoleucine 1160 is highly conserved, particularly in mammals

In HGMD database, 708 *COL3A1* mutations, mostly missense, are listed. Several missense mutations associated with vEDS have been described in the 10‐amino acid regions upstream and downstream isoleucine 1160 (Figure 1), suggesting the presence of a potential hot‐spot region at this level. This residue of isoleucine is highly conserved, particularly in mammals (Figure 2).

Further workup was aimed at investigating the pathogenicity of the variant.

The segregation analysis, performed only in the mother (father died at 59 years of lung cancer), was negative, partially supports the de novo origin of the variant.

**FIGURE 3:**
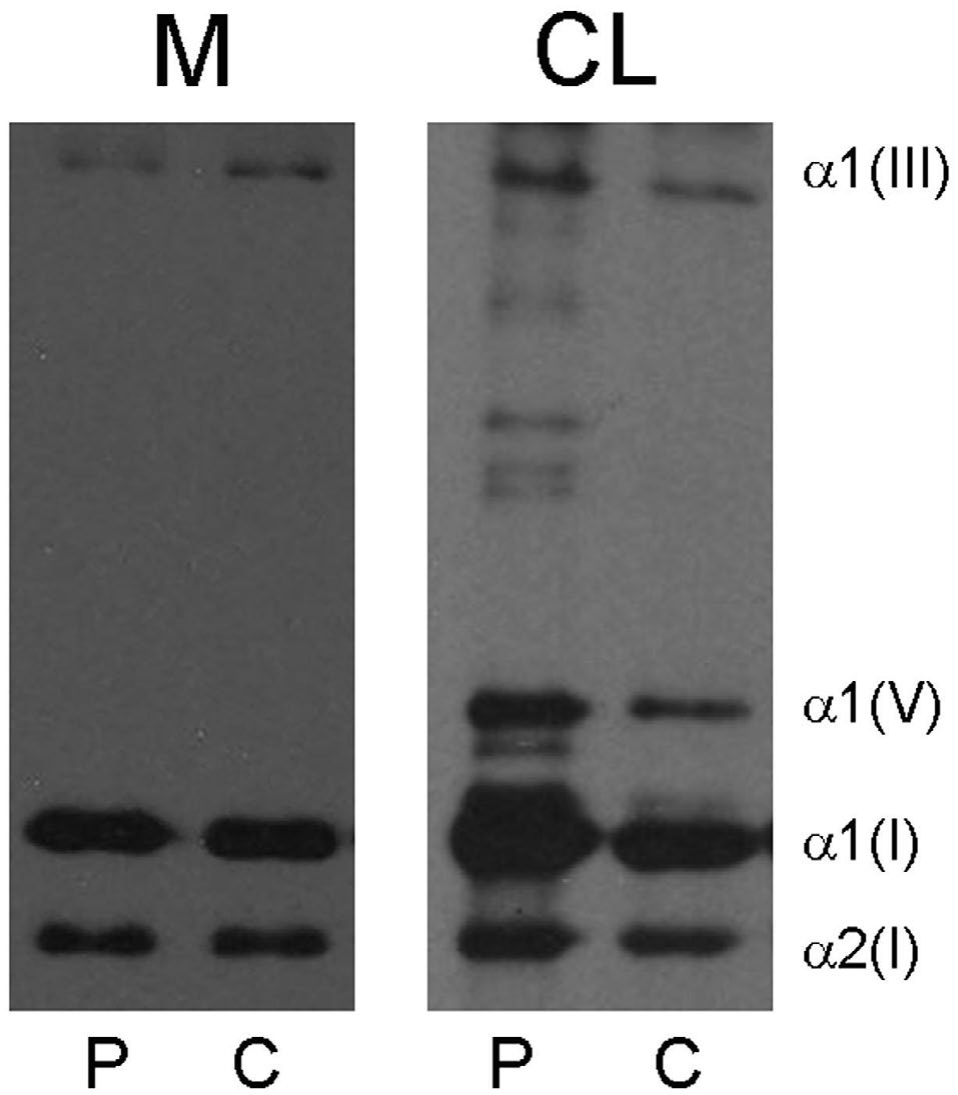
Six percent sodium dodecyl sulfate‐polyacrylamide gel electrophoresis of pepsin‐treated collagen secreted by fibroblasts (M) and retained in cell layer (CL) of a patient affected by vEDS (P) and an age‐matched control (C). In the first and in the third lanes a delay in the electrophoretic mobility of α1(III) chain is observed. Furthermore, a decreased amount of α1(III) chain can be detected

Afterward, a skin sample of the patient was achieved to perform a functional study on the fibroblasts. The fibroblasts cultures were grown and maintained in Dulbecco modified Eagle's medium (DMEM) with 10% fetal calf serum. Type I, III, and V collagens were purified from medium and cell layer as described by Valli et al. (1991) and then separated by gel electrophoresis. Gels were processed for fluorography using standard procedures and submitted to quantitation by using the program Image J released from National Institute of Health, USA. The reference range of type III/I collagen ratio has been determined by measuring this ratio in samples collected from healthy subjects over a period of 3 years. This analysis showed delayed electrophoretic mobility of type III collagen (Figure 3). A decreased amount of α1(III) chain, as confirmed by the densitometric scan of the fluorogram, was also detected.

**FIGURE 4:**
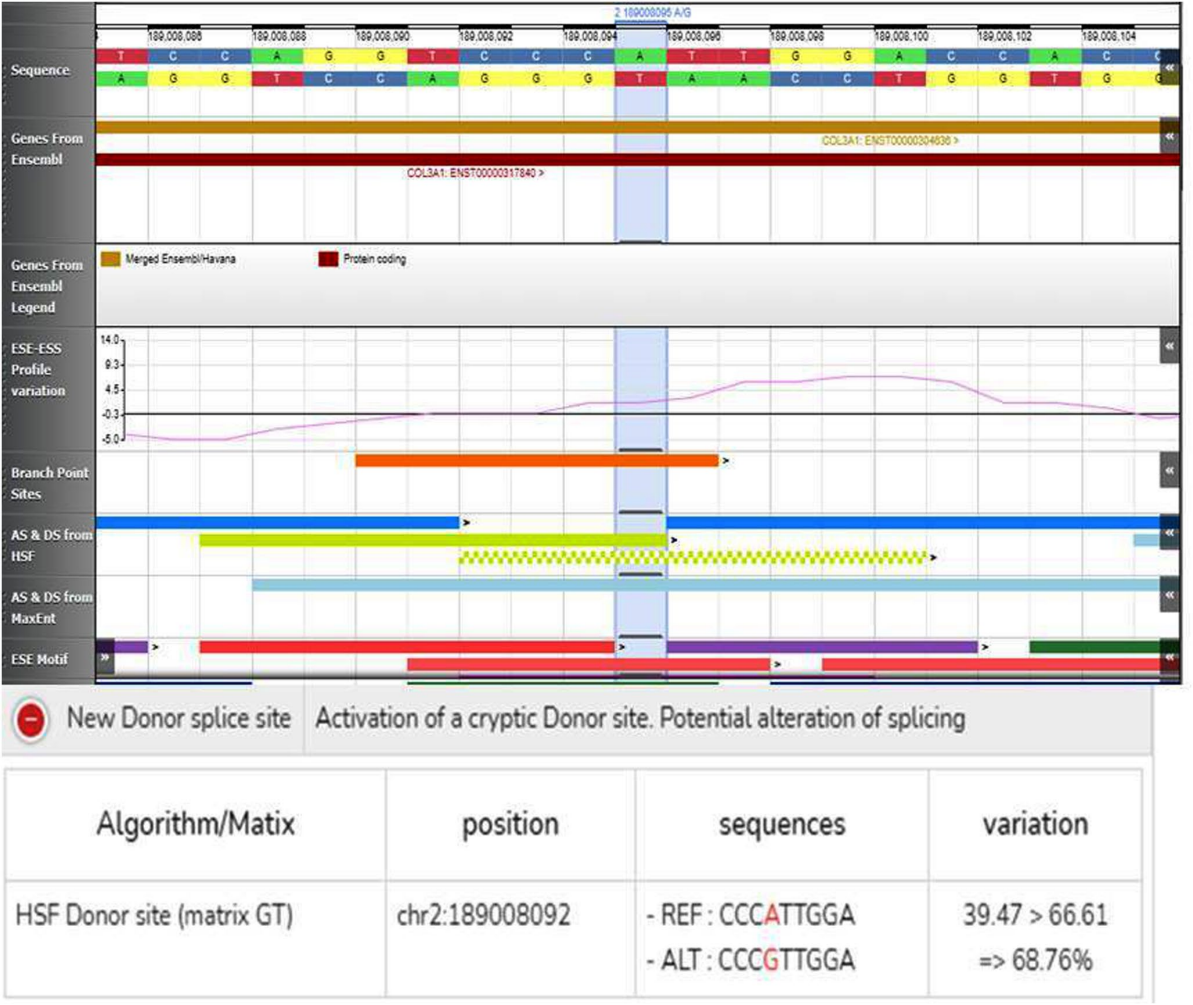
Computational analysis of the c.3478A>G variant in *COL3A1* (NM_000090.3) through “Human Splicing Finder”: the A>G transition potentially leads to the activation of a cryptic donor splice site

In the hypothesis that the electrophoretic abnormalities were due to post‐transcriptional modifications of the mRNA we analyzed the mutation through “Human Splicing Finder” (Figure 4), which predicted the creation of a cryptic donor splice site (CCCGTTGGA).

The employment of this cryptic site may result in the synthesis of shortened chains with consequent alteration at a post‐translational level, especially in the trimer folding process. Although the prediction made by the software was weak, alternative splicing have been considered as an additional cause of the electrophoretic delay and further mRNA analysis on fibroblasts of the patient has been performed.

Further mRNA analysis on fibroblasts of the patient shows no modifications on mRNA length through electrophoresis and Sanger analysis revealing no splicing defects.

Nevertheless, adding the finding of the protein study as a parameter in the computational analysis, the variant can be classified as likely pathogenic according to ACMG2015 criteria.

**FIGURE 5:**
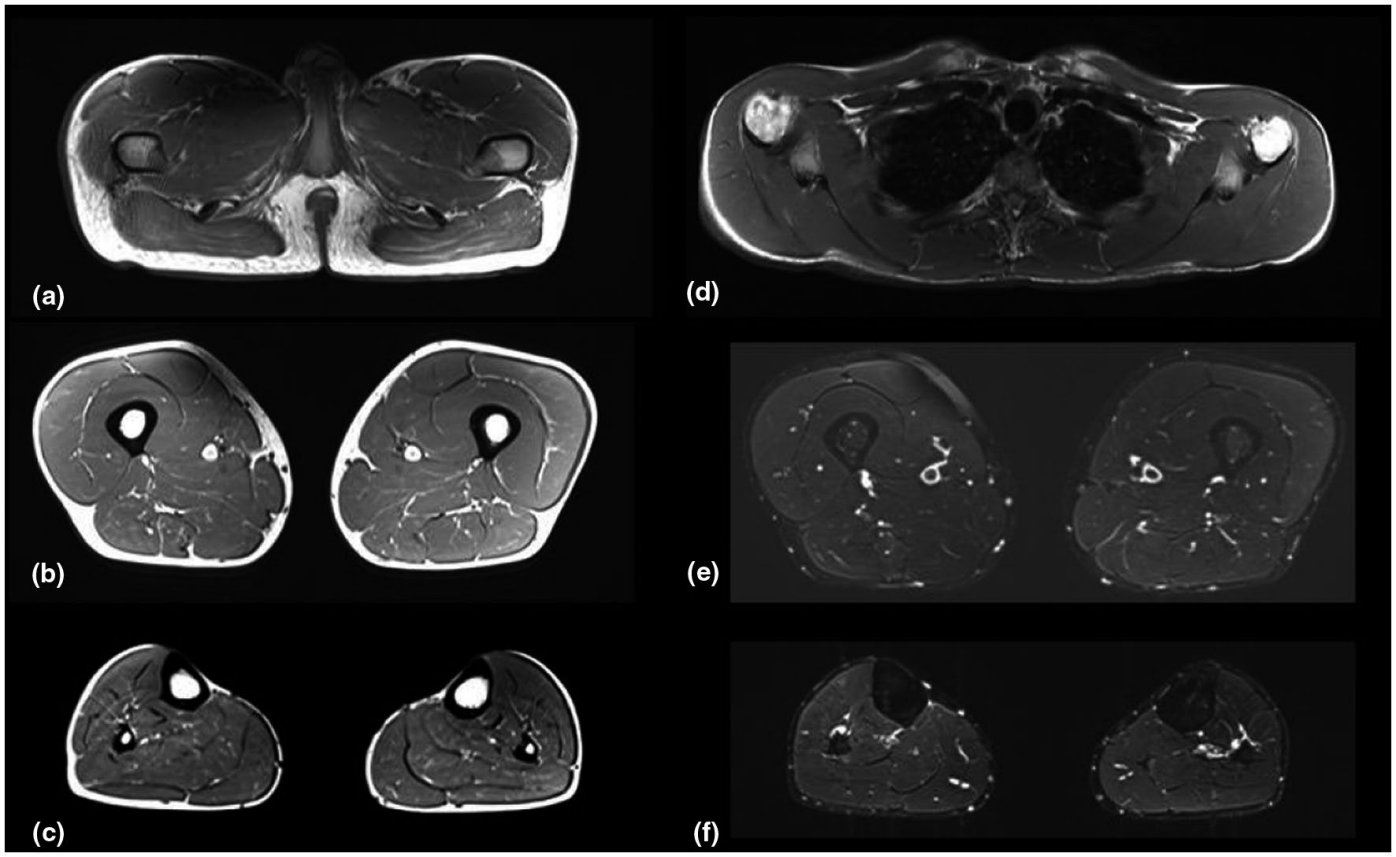
(a–f). Axial T1‐SE (a,b,c,d) and T2‐STIR sequences (e and f) of the pelvic girdle (a), shoulder girdle (d), thigh (b and e), leg (c and f), bilaterally. Muscle trophism and signal are normal at the level of the thigh (b) and shoulder girdle (d), whereas slight hyperintensity can be detected at the level of glutei maximi (Mercuri score 1) (a) and of the muscles of the posterior compartment of the legs (c). No muscle edema could be detected in the inferior limbs (e and f)

A muscular MRI of neck, upper, and lower limbs with axial and coronal T1 and T2‐STIR weighted sequences (Figure 5) was performed to seek the consequences of type III collagen abnormality on the muscle tissue level. This investigation did not show a selective pattern of muscle involvement, but only diffuse slight signs of fat substitution, mainly in gluteus and soleus muscles, bilaterally. No signs of fat substitution in the other muscle districts examined were reported, neither muscle edema could be detected.

## 3 DISCUSSION

Ehlers–Danlos syndrome (EDS) is a heterogeneous group of heritable connective tissue disorders with 13 clinically distinct subtypes (Malfait et al., 2017). Vascular EDS (vEDS) is caused by heterozygous pathogenic variants in *COL3A1* gene (Pepin et al., 2000). *COL3A1* encodes for the α1 chain of type III collagen, which assembles into a homotrimer to form type III collagen molecules. Since it is a primary structural component of blood vessels and hollow organs, reduced or abnormal type III collagen leads to an increased risk of spontaneous artery or organ ruptures. For this reason, vEDS is generally considered the most severe form of EDS and it is associated with a decreased life expectancy (Oderich et al., 2005).

Although tendon/muscle ruptures are included in the minor diagnostic criteria of vEDS (Malfait et al., 2017), predominant or exclusive musculoskeletal involvement is not a common clinical presentation of the disease. Therefore, in absence of family history, the diagnosis of vEDS can be challenging in a patient who only presents muscle ruptures, even if they are multiple and not associated with any major trauma.

When faced with this phenotype, Marfan syndrome and collagenopathy should be both included in the differential diagnosis, since they share similar clinical characteristics.

Indeed, after the exclusion of Marfan syndrome (more frequently associated with musculoskeletal involvement) through the assessment of Marfan Systemic Score and/or *FBN1* analysis, clinicians should consider collagenopathy in its different, and perhaps atypical, forms.

A musculoskeletal involvement of different degrees characterizes all the EDS subtypes with a less severe impairment in the vEDS than in the other subtypes (Malfait et al., 2017).

vEDS was the EDS subtype which could fit in the clinical features of the patient herein described. Indeed, he presented mitral regurgitation (if progressive, it is considered a major criterium for this EDS subtype) together with other minor criteria such as pectus excavatum, pes planus, and joint dislocations. Being the most severe form of EDS, vEDS is generally ruled out in the diagnostic pathway. However, given the extremely variable phenotype, vEDS should be considered even if only one minor criterium (muscle ruptures) is present.

Therefore, we suggest that, even when suspecting a specific subtype of EDS, clinicians should consider the analysis of a next‐generation sequencing gene panel including all forms of collagenopathy.

With our report, we describe a novel variant of *COL3A1* in a non‐glycine codon, whose pathogenic role has been supported by collagen analysis.

In literature, most mutations described involve glycine residues which are essential for triple helix of type III collagen structure. In the last few years mutations in codons different from glycine have been described associated with various EDS phenotypes (Ghali et al., 2019). Several mutations in different domains of the protein have been described mostly associated with vascular and classical EDS subtypes. Familial and sporadic cases have been reported with inter‐familial variability. The prevalence of vEDS phenotype associated to COL3A1 and described in the literature could possibly be due to an enrollment bias; indeed, all the *COL3A1* analyses have been performed on well‐defined EDS subtypes patients (see Table 1 for further details).

**TABLE 1:** COL3A1 no‐glycine substitutions and relative phenotype as described in literature

Mutation	References	Sex	Age	Phenotype	Familiarity	Relatives
E241K	Angwin et al., (2020)	F	30	vEDS	sporadic	
E241K	Ghali et al., (2019)	F	33	cEDS	sporadic	
E241K	Ghali et al., (2019)	M	54	cEDS	sporadic	
R271Q	Frank et al., (2015)	F	45	vEDS	sporadic	
R271Q	Frank et al., (2015)	F	47	vEDS	sporadic	
N389Y	Frank et al., (2015)	F	44	vEDS	familial	Family 1
N389Y	Frank et al., (2015)	F	49	vEDS	familial	Family 2
N389Y	Frank et al., (2015)	F	70	vEDS	familial	Family 3
E682K	Ghali et al., (2019)	M	50	vEDS/cEDS	familial	Family 4
E682K	Ghali et al., (2019)	M	73	EDS	familial	Family 4
E682K	Ghali et al., (2019)	F	65	EDS	familial	Family 4
E682K	Ghali et al., (2019)	M	74	EDS	familial	Family 4
E682K	Ghali et al., (2019)	M	3	EDS	familial	Family 4
E682K	Ghali et al., (2019)	F	35	cEDS	familial	Family 4
E682K	Ghali et al., (2019)	M	44	cEDS	familial	Family 4
E682K	Ghali et al., (2019)	F	8	EDS	familial	Family 4
E682K	Ghali et al., (2019)	F	5	EDS	familial	Family 4
E682K	Ghali et al., (2019)	M	37	vEDS	sporadic	
E682K	Ghali et al., (2019)	F	22	cEDS	familial	Family 5
E682K	Ghali et al., (2019)	F	14	EDS	familial	Family 5
E682K	Ghali et al., (2019)	M	50	cEDS	familial	Family 5
P806L	Weerakkody et al., (2016)	F	38	vEDS / hEDS overlap	sporadic	
R1082Q	Frank et al., (2015)	M	60	vEDS	sporadic	
E1171K	Frank et al., (2015), Henneton et al., (2019), Ghali et al., (2019)	M	22	vEDS	sporadic	
E1171K	Angwin et al., (2020)	M	25	vEDS	sporadic	
E1171K	Angwin et al., (2020)	F	49	vEDS	sporadic	
E1171K	Ghali et al., (2019)	F	53	cEDS	familial	Family 6
E1171K	Ghali et al., (2019)	M	27	cEDS	familial	Family 6
A1203T	Frank et al., (2015)	F	54	vEDS	sporadic	
P1258S	Frank et al., (2015)	F	51	vEDS	familial	Family 7
P1258S	Frank et al., (2015)	M	21	asymptomatic relative	familial	Family 7
A1259T	Frank et al., (2015)	F	43	vEDS	familial	Family 8
P1270T	Frank et al., (2015)	F	17	vEDS	familial	Family 9
P1270T	Frank et al., (2015)	F	45	asymptomatic relative	familial	Family 9
K1273R	Frank et al., (2015)	F	48	vEDS	familial	Family 10
K1273R	Frank et al., (2015)	F	55	vEDS	familial	Family 10
K1273R	Frank et al., (2015)	F	45	vEDS	sporadic	
D1288V	Kerwin et al., (2008)	—	—	vEDS	—	
K1313R	Stembridge et al., (2015)	F	30	benign hypermobility	familial	Family 11
K1313R	Frank et al., (2015)	F	38	vEDS	sporadic	
R1432L	Stembridge et al., (2015)	M	39	vEDS	familial	Family 12
R1432L	Stembridge et al., (2015)	F		hEDS	familial	Family 12
P1440L	Weerakkody et al., (2016)	F	26	vEDS	sporadic	
P1440L	Stembridge et al., (2015)	F	27	vEDS	sporadic	
P1440L	Morissette et al., (2014)	F	41	vEDS	sporadic	

Abbreviations: cEDS, classical EDS; EDS, not specified EDS subtype; hEDS, hypermobile EDS; vEDS, vascular EDS.

Our findings broaden the phenotypic spectrum of vEDS, reporting a patient with predominant musculoskeletal and very mild cardiac involvement. The reaching of a definite diagnosis is very important for patients, particularly those with atypical clinical presentations, to avoid inappropriate therapies and to establish a correct follow‐up. Moreover, a correct diagnosis has significant relevance in genetic counseling giving a correct recurrence and procreative risk to the patient and family members.

## CONFLICT OF INTEREST

The authors have no actual or potential conflicts of interest. The authors state that no approvals by ethics committee are required for the issue of this report.

## AUTHOR CONTRIBUTIONS

Federica Ruscitti: conceptualization, design analysis, planning, conduct, data analysis, manuscript preparation, review of literature. Lucia Trevisan: conceptualization, design analysis, planning, conduct, data analysis, manuscript preparation, review of literature. Giulia Rosti: conduct, manuscript preparation, review of literature. Fabio Gotta: conduct, manuscript preparation. Annalia Cianflone: conduct, manuscript preparation. Alessandro Geroldi: data analysis, manuscript preparation, review of literature. Paola Origone: data analysis, manuscript preparation, review of literature. Anna Pichiecchio: data analysis, review preparation. Simona Viglio: data analysis, review preparation. Maria Iascone: conceptualization, data analysis, design analysis, planning. Paola Mandich: conceptualization, design analysis, planning, conduct, manuscript preparation.

## ETHICAL COMPLIANCE

Informed consent of the patient and family members for the molecular test has been stated from a medical geneticist.

## Data Availability

The datasets generated during the current study are available from the corresponding author on reasonable request.
